# Targeted HPLC-UV Polyphenolic Profiling to Detect and Quantify Adulterated Tea Samples by Chemometrics

**DOI:** 10.3390/foods12071501

**Published:** 2023-04-03

**Authors:** Thom Romers, Javier Saurina, Sònia Sentellas, Oscar Núñez

**Affiliations:** 1 Department of Chemical Engineering and Analytical Chemistry, Universitat de Barcelona, Martí i Franquès 1-11, E08028 Barcelona, Spain; 2 Research Institute in Food Nutrition and Food Safety, Universitat de Barcelona, Av. Prat de la Riba 171, Edifici Recerca (Gaudí), E08921 Santa Coloma de Gramenet, Spain; 3 Serra Húnter Fellow, Departament de Recerca i Universitats, Generalitat de Catalunya, Via Laietana 2, E08003 Barcelona, Spain

**Keywords:** tea, chicory, HPLC-UV, polyphenols, chemometrics, food authentication, food safety

## Abstract

Tea can be found among the most widely consumed beverages, but it is also highly susceptible to fraudulent practices of adulteration with other plants such as chicory to obtain an illicit economic gain. Simple, feasible and cheap analytical methods to assess tea authentication are therefore required. In the present contribution, a targeted HPLC-UV method for polyphenolic profiling, monitoring 17 polyphenolic and phenolic acids typically described in tea, was proposed to classify and authenticate tea samples versus chicory. For that purpose, the obtained HPLC-UV polyphenolic profiles (based on the peak areas at three different acquisition wavelengths) were employed as sample chemical descriptors for principal component analysis (PCA) and partial least squares-discriminant analysis (PLS-DA) studies. Overall, PLS-DA demonstrated good sample grouping and discrimination of chicory against any tea variety, but also among the five different tea varieties under study, with classification errors below 8% and 10.5% for calibration and cross-validation, respectively. In addition, the potential use of polyphenolic profiles as chemical descriptors to detect and quantify frauds was evaluated by studying the adulteration of each tea variety with chicory, as well as the adulteration of red tea extracts with oolong tea extracts. Excellent results were obtained in all cases, with calibration, cross-validation, and prediction errors below 2.0%, 4.2%, and 3.9%, respectively, when using chicory as an adulterant, clearly improving on previously reported results when using non-targeted HPLC-UV fingerprinting methodologies.

## 1. Introduction

Food integrity, quality and safety are of paramount importance to society. The selection of a specific food product is based on consumers’ benefit perception, which depends on a great variety of properties [1]. Food origin, agricultural practices and health benefits (functional foods) are among the attributes being considered by society when selecting food products. In general, the information provided in the food labels makes consumers select one or another product, and thus, in order to protect consumers, governments and regulatory bodies establish rigorous legislation on food labeling [2,3]. Nevertheless, despite these legal requirements, food production is within a globalized world and the food chain is extremely complex, making possible food manipulation and adulteration practices aiming at obtaining an illicit economic profit. Food fraud involves the deliberate addition, substitution or misrepresentation of a foodstuff, ingredient or food packaging [4]. Within this context, food adulteration is carried out for an economic profit by either increasing the volume, masking the occurrence of lower-quality components, or replacing authentic substances. These practices may have not only economic consequences, but also important health issues when prohibited chemicals are employed to mask organoleptic properties. In some cases, the adulterant may even contribute to trigger strong allergic episodes, making food adulteration control an important issue to guarantee food safety. Therefore, developing non-expensive, simple and feasible analytical methods able to assess food authenticity and prevent frauds is required.

Tea is an aromatic drink obtained from the leaves of the *Camellia sinensis* plant (an evergreen shrub native to East Asia and China). The characteristic aroma and flavor of tea infusions, and their numerous recognized health-beneficial attributes (e.g., antioxidant, antimicrobial, anticarcinogenic, and neuroprotective properties, among others), are responsible for the high consumption of this drink on a daily basis [5,6,7,8,9,10]. Although a great variety of tea can be found in the market, five types, according to their fermentation procedure, are the most common: black, green, oolong, red (Pu-erh), and white. Green and white teas are not fermented varieties. Green tea is steamed or pan-fired immediately after harvest to stop their oxidation, while white tea is packaged after drying with minimum pre-processing. White tea is maybe the most expensive and appreciated by the consumers, produced from the very first buds and tips of the plant. Black tea is one of the most traditional varieties, where full oxidation was allowed. Red (Pu-erh) teas are obtained from a specific variant of the tea plant (*Camellia sinensis* var. assamica), produced only in the region of Yunnan (China). This variety is processed in humid conditions to allow bacteria composting action. Finally, oolong tea is a controlled fermented variety where oxidation is limited to 10–70%.

Tea is one of the most adulterated drinks, with cereal starch, legume husks and chicory as common adulterants of tea [11,12]. In this work, chicory (*Chicorium intybus*), a perennial herbaceous plant belonging to the Astareceae family, was studied as a tea adulterant as its use in tea must be completely banned when non-declared because of possible health effects [13,14].

The classification and authentication of teas has been addressed either by non-targeted or targeted analysis, together with multivariate chemometric methodologies. For example, a targeted determination of metal elements by inductively coupled plasma-mass spectrometry (ICP-MS) followed by linear discriminant analysis was described to assess the region of production of several green teas from China [15]. Polyphenolic and phenolic compounds have also been employed as targeted tea markers to trace Keemun black tea production regions [16]. Fingerprinting strategies (non-targeted methods), based on monitoring instrumental responses (with the aim of detecting as many components as possible), but not requiring identification of the compounds responsible for those signals, have also been described to solve tea authenticity [17,18,19,20,21]. For example, non-targeted high-performance liquid chromatography with ultraviolet (HPLC-UV) and fluorescence (HPLC-FLD) detection followed by partial least squares-discriminant analysis (PLS-DA) were developed to classify and authenticate tea versus chicory [22]. Recently, we proposed a high-throughput non-targeted flow injection analysis-mass spectrometry (FIA-MS) methodology to classify and authenticate tea versus chicory [23]. Although, overall, both HPLC-UV-FLD and FIA-MS fingerprinting methodologies were able to discriminate chicory from any tea variety with acceptable classification rates, prediction errors for adulterated tea samples with chicory using partial least squares (PLS) regression were still very high for some tea varieties, especially for white and black teas [22,23].

In the present contribution, a simple HPLC-UV polyphenolic profiling method followed by chemometrics was developed to detect and quantify adulterated tea samples (using chicory as adulterant). With this in mind, seventeen phenolic compounds, typically described in tea samples, were selected. Samples were analyzed by C18 reversed-phase HPLC-UV, and peak areas of the identified compounds were used as chemical descriptors for chemometrics. A total of 120 samples including five tea varieties and chicory were analyzed for classification using principal components analysis (PCA) and PLS-DA. Then, adulteration cases based on the adulteration of each tea variety with chicory, as well as the adulteration of red tea with oolong tea, were processed by PLS regression to study the capability of the proposed HPLC-UV polyphenolic profiling method for the detection and quantitation of adulterant levels in tea extracts.

## 2. Materials and Methods

### 2.1. Chemicals

Acetonitrile (UHPLC supergradient ACS quality) from PanReac AppliChem (Barcelona, Spain), and formic acid (≥98%) from Sigma-Aldrich (St. Louis, MO, USA) were used. Purified water (Elix 3 coupled to a Milli-Q system from Millipore Corporation, Bedford, MA, USA) was employed. A commercially available mineral water obtained from Eroski (Elorrio, Spain) was used for tea and chicory brewing.

Seventeen polyphenolic and phenolic acid chemicals were employed, all of them of analytical grade. Gallic, caffeic, ferulic, vanillic, sinapic, syringic, *p*-coumaric, 4-hydroxybenzoic and 3,4-dihydroxybenzoic acids, and quercetin, (−)-epicatechin, rutin, apigenin, and kaempferol were purchased from Sigma-Aldrich; (+)-catechin and myricetin from Fluka (Madrid, Spain); and (−)-epigallocatechin from Biosynth Carbosynth (Berkshire, United Kingdom).

Stock standard solutions of the targeted polyphenolic chemicals (ca. 1000 mg L^−1^) were prepared in methanol or DMSO (depending on the compound). Working solutions were obtained by the corresponding dilution with Milli-Q water of the stock standards.

### 2.2. Instrumentation

Chromatographic separation was performed on a Kinetex^®^ C18 column (100 × 4.6 mm i.d., 2.6 µm particle size), equipped with a C18 guard-column (2 mm × 4.6 mm id., 2.6 µm particle size), provided by Phenomenex (Torrance, CA, USA) using a HPLC instrument from Agilent HPLC 1100 Series (Waldbronn, Germany) equipped with a G1312A binary pump, a WPALS G1367A automatic sample injector, a G1315B diode-array detector, and a PC with the Agilent Chemstation software (2001–2012 version).

A gradient elution separation was proposed for the determination of polyphenols and phenolic acids, as well as other components of the analyzed samples; solvent A was 0.1% formic acid in water and solvent B was acetonitrile. Elution program: 0–1 min from 5–10% solvent B; 1–4 min from 10–16% solvent B; 4–8 min, isocratic at 16% solvent B; 8–8.5 min, from 16–25% solvent B; 8.5–13.5 min, from 25–60% solvent B; 13.5–16 min, from 60–100% solvent B; 16–16.5 min, isocratic at 100% solvent B; 16.5–16.6 min, back to initial conditions at 5% solvent B; and 16.6–22 min isocratic at 5% solvent B for column re-equilibration. Other conditions: 800 µL min^−1^ as mobile phase flow rate; 5 µL of injection volume; and room temperature for column separation. Ultraviolet-visible (UV-vis) acquisition was performed at 280, 325 and 370 nm, where the targeted polyphenolic compounds presented maximum UV-vis absorption spectrum values, and due to the fact that they resulted to be the most discriminant ones for classification purposes based on previous studies with the kind of samples analyzed.

### 2.3. Samples

A total of 100 teas of different varieties including white, black, green, red and oolong extracts (20 samples/variety), as well as 20 chicory extracts, obtained from different markets in Barcelona (Spain), were employed. The description of the samples (commercial brands and countries of production) and the number of lots employed for each sample is provided in Table S1 (Supplementary Materials).

Tea and chicory extracts were obtained following a previously established procedure [22,23]. Briefly, ca. 0.5 g of tea/chicory sample were placed in a 50 mL PTFE centrifuge tube (Serviquimia, Barcelona, Spain) and extracted with 25 mL of hot mineral water by vigorously shaking (1 min) in a Vortex (Stuart, Stone, UK). The extract was centrifuged (5 min, 3500 rpm) in a Rotanta 460 RS centrifuge (Hettich, Tuttlingen, Germany), filtered through 0.45 µm syringe nylon membrane filters (discarding the first mL), and kept at 4 °C in glass injection vials until their HPLC-UV analysis. In addition, a quality control (QC) composed sample was prepared by mixing 50 µL of each tea/chicory aqueous extract. This QC solution is used to ensure the repeatability of the HPLC-UV method and the robustness of the obtained chemometric results.

### 2.4. Data Analysis

Samples were randomly analyzed with the HPLC-UV methodology. In addition, a QC, a polyphenolic standard mixture, and a blank (a mineral water) were analyzed after every 10 samples. Polyphenolic and phenolic acid peak areas detected at the three registered acquisition wavelengths were then measured and used to generate the profiling data matrices, which were then submitted to PCA, PLS-DA, and PLS regression methods using SOLO 8.6 chemometric software from Eigenvector Research (Manson, WA, USA). The theoretical background of these methods is addressed in reference [24]. For all the chemometric methods, the X-data matrix of response variables consisted of the obtained HPLC-UV polyphenolic profiles (i.e., the peak areas detected at the three registered acquisition wavelengths for the 17 polyphenolic and phenolic acids employed as targeted compounds). The Y-data matrix defines each sample class (tea variety or chicory) in PLS-DA, whereas it defines each adulterant percentage in PLS. To provide the same weight to each variable, in order to remove differences in magnitude and amplitude scales, the obtained data were autoscaled. Latent variables (LVs) were established as the first significant minimum point of the cross-validation (CV) error from a Venetian blind approach.

To validate the obtained PLS-DA models, 60% of the samples (randomly selected) were employed for calibration while the other 40% were used for prediction. To evaluate the predictive performance of the classification models, the sensitivity was calculated as TP/(TP + FN), the specificity as TN/(TN + FP), and the accuracy as (TP + TN)/TS, all of them expressed as a percentage, with TP being the number of positive samples correctly assigned to the class, TN the number of negative samples correctly assigned (i.e., not belonging to the class), FN the number of false negatives incorrectly assigned as not belonging to the class, FP the number of false positives incorrectly assigned to the class, and TS the total number of samples.

Adulteration cases studied by PLS consisted of each one of the five different tea samples adulterated with chicory, as well as red tea adulterated with oolong tea. For that purpose, the PLS calibration set comprises adulteration levels of 0, 20, 40, 60, 80 and 100%, whereas adulteration levels of 15, 25, 50, 75 and 85% were used for validation. Each one of the adulteration levels employed was done by quintuplicate. For the preparation of these adulterations, five pooled tea samples were prepared for each tea variety by mixing 10 different samples (different origins) of the same variety, and each pooled tea sample was then adulterated with a different chicory sample. Thus, the five replicates for each adulteration level were obtained using different combinations of tea/chicory samples. Table S2 (Supplementary Materials) shows the information on how the extracts were blended for the PLS adulteration studies. The same strategy was employed when in the adulteration study of red tea with oolong tea. Moreover, as QC extract, an additional 50% adulteration level was employed. Again, all adulterated samples were randomly analyzed with the proposed HPLC-UV methodology, and the QC, a polyphenolic standard mixture, and a blank (a mineral water sample) were analyzed every ten samples.

## 3. Results and Discussion

In previous publications, we evaluated the suitability of non-targeted fingerprinting strategies based on HPLC-UV and HPLC-FLD [22] and FIA-MS [23] for the characterization and classification of tea and chicory samples. Although acceptable results were obtained to discriminate tea varieties against chicory, no discrimination was accomplished between the different tea varieties under study. In addition, prediction errors higher than 20% were obtained in some cases when HPLC-UV or HPLC-FLD fingerprints were employed as sample chemical descriptors to detect and quantify chicory as an adulterant in tea extracts, especially for some tea varieties such as white, black or green teas. This finding was attributed to the similarity on their chromatographic fingerprints. Although results improved with FIA-MS fingerprints, being also an advantageous technique due to its high-throughput performance, prediction errors were still relatively high (>12% in most of the evaluated cases). In addition, mass spectrometry is a more expensive technique and requires specialized technicians, circumstances that cannot be afforded in many control laboratories, especially in some tea-producer countries. To improve the previously published results, a simple targeted HPLC-UV polyphenolic profiling method is developed and its application to classify tea and chicory extracts, as well as detect chicory fraud, is evaluated.

### 3.1. Polyphenolic Separation and Polyphenolic Profiles of Pure Tea and Chicory Extracts

A total of 17 flavonoids and phenolic acids typically occurring in tea samples were selected as the targeted compounds [25]. As reported in the literature, the chromatographic separation of polyphenols can be accomplished by reversed-phase mode. In the present work, a Kinetex^®^ C18 porous-shell reversed-phase column using gradient elution with acetonitrile and 0.1% formic acid as mobile phase components (flow rate 800 µL min^−1^) was employed (gradient program described in Section 2.2). Figure S1 (Supplementary Materials) shows the HPLC-UV (registered at 280 nm) of a mixture of the 17 targeted phenolic chemicals at a concentration of 20 mg L^−1^. Although baseline separation was not accomplished for all compounds (four pairs present coelution), as a compromise between analysis time and peak resolution, the obtained chromatographic separation was considered acceptable for the intended purpose of this work. It should be taken into account that despite the presence of some polyphenolic coelutions, the aim of the present work is not the quantification of phenolic compounds but the monitoring of their peak signals as potential descriptors to accomplish the classification and authentication of the analyzed samples. In those coelution cases where the signal of both compounds cannot be distinguished based on their different response at the three-acquisition wavelength, it must be considered that the signal peak area employed for further studies will include the contribution of both coeluting compounds.

**Figure 1:**
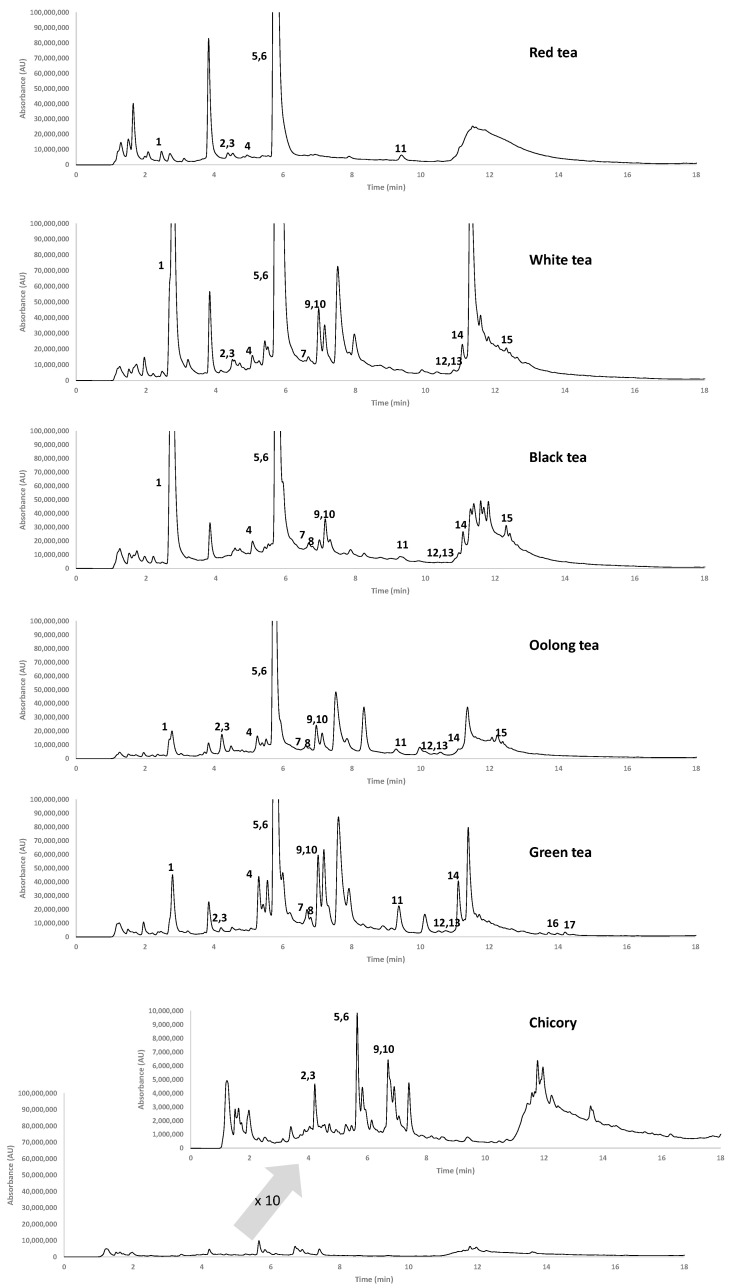
HPLC-UV chromatograms recorded at 280 nm for selected samples for each tea variety and a chicory extract. Peak identification: 1, gallic acid; 2, protocatechuic acid; 3, quercetin; 4, (−)-epigallocatechin; 5, (+)-catechin; 6, 4-hydroxybenzoic acid; 7, vanillic acid; 8, caffeic acid; 9, syringic acid; 10, (−)-epicatechin; 11, *p*-coumaric acid; 12, ferulic acid; 13, sinapic acid; 14, rutin; 15, myrcetin; 16, apigenin; 17, kaempferol.

Tea and chicory samples were extracted as described in Section 2.3, and the extracts analyzed with the HPLC-UV method. Three acquisition wavelengths (280, 325 and 370 nm) were selected to cope with the detection of different families of polyphenolic compounds. The tentative identification of polyphenols was based on comparing both retention times and UV spectra of the sample chromatographic peaks with those of pure standards (in those cases with coelutions, contribution of both polyphenolic coeluting compounds was considered). Figure 1 depicts characteristic chromatograms (at 280 nm) for each tea variety and for chicory. As can be seen, the most notable difference is the 10-fold higher intensity of tea extract fingerprints in comparison to those of chicory samples. In addition, different chromatographic profiles were observed for each tea variety and chicory, pointing out differences also in the occurrence of polyphenolic compounds. The gallic acid signal is particularly intense in white and black teas, while (+)-catechin and 4-hydroxybenzoic are predominant among the analyzed compounds in all tea extracts.

The differences observed in the chromatograms, not only on the targeted polyphenolic compounds detected but also on their corresponding peak signals, and the fact that the obtained HPLC-UV polyphenolic profiles seem to be reproducible for all the samples within the same class variety, suggested that the targeted HPLC-UV polyphenolic profiling data could be proposed as sample chemical descriptors to study the classification of tea and chicory by chemometrics.

### 3.2. Sample Characterization by Principal Component Analysis

The signal peak areas of the 17 targeted flavonoid and phenolic acid compounds at the three monitored acquisition wavelengths in all the analyzed tea and chicory samples were employed to build the X-data matrix for chemometrics. The resulting data matrix had a dimension of 133 × 30 (tea/chicory/QC samples × detected polyphenolic peak areas at three wavelengths). It should be commented that some compounds are not detected at some of the registered acquisition wavelengths. This matrix was first subjected to PCA to explore the sample distribution and evaluate data repeatability, as well as the robustness from the QCs behavior.

**Figure 2:**
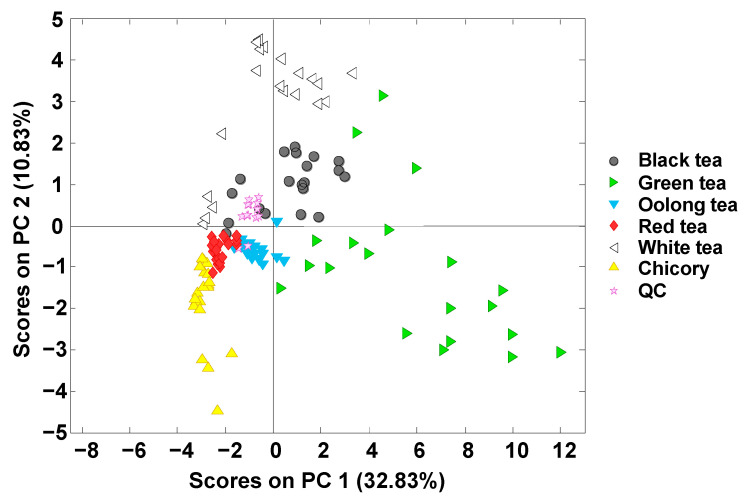
PCA score plot of PC1 versus PC2 when using targeted HPLC-UV polyphenolic profiles as sample chemical descriptors.

The score plot of PC1 versus PC2 is depicted in Figure 2. PC1 × PC2 retained 43.66% of the sample variance observed. As can be seen, QC samples, which were analyzed at the beginning and every ten samples, appeared well grouped, ensuring the reproducibility and robustness of the method since no sequence drift was encountered. QC repeatability as percentage of relative standard deviation (%RSD) of the total signal registered was 5.4%.

Focusing on the analyzed samples, they are grouped based on their variety. Chicory samples appeared at the left-bottom area of the plot (showing negative values for both PC1 and PC2), close to red tea samples. This last group, together with the oolong samples, are grouped compactly, and mainly presenting negative values for PC1 and PC2. In contrast, white and black tea samples are mainly characterized as presenting positive PC2 values, while green tea samples have positive PC1 values, appearing mainly in the right area of the plot. In any case, chicory samples are clearly discriminated from all the tea varieties (although located close to red tea ones, as previously commented). A noteworthy result is that acceptable sample discrimination was also observed with the five tea varieties when using the polyphenolic profiles based on the peak areas of seventeen polyphenols at three different acquisition wavelengths. This good sample discrimination was not observed in previous studies when either non-targeted HPLC-UV-FLD or FIA-MS fingerprints were employed [22,23], where overlapping between tea varieties, as well as with some chicory samples, was observed.

### 3.3. Sample Classification by PLS-DA

**Figure 3:**
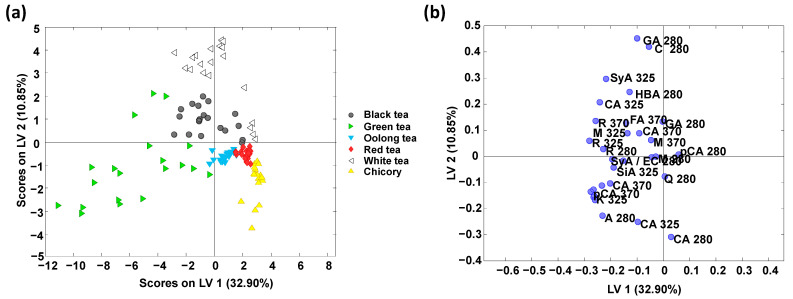
PLS-DA score plot (**a**) and loading plot (**b**) of LV1 versus LV2 when using targeted HPLC-UV polyphenolic profiles as sample chemical descriptors for the classification of tea and chicory samples. Six LVs were employed to build the model. Compound identification: gallic acid, GA; protocatechuic acid, PA; quercetin, Q; (−)-epigallocatechin, EGC; (+)-catechin, C; 4-hydroxybenzoic acid, HBA; vanillic acid, VA; caffeic acid, CA; syringic acid, SyA; (−)-epicatechin, EC; *p*-coumaric acid, pCA; ferulic acid, FA; sinapic acid, SiA; rutin, R; myrcetin, M; apigenin, A; kaempferol, K.

The X-data matrix (see Section 3.2) without considering the QCs (matrix dimension 120 × 30, tea/chicory samples × polyphenolic peak areas) was subjected to supervised PLS-DA for classificatory studies. In this case, a Y-data matrix including the sample classes (black, white, green, red, and oolong teas, and chicory) was employed. Figure 3 shows the obtained PLS-DA (a) score and (b) loading plots of LV1 versus LV2 when using the obtained polyphenolic profiles as sample chemical descriptors. A similar discrimination among classes to the one observed by PCA was obtained, although with a different spatial distribution of the sample groups within the plot. In any case, samples are clearly grouped according to tea variety and chicory (the studied adulterant) classes.

A paired PLS-DA model was built to classify the tea varieties analyzed versus chicory. With this in mind, 60% of the samples randomly selected were used for calibration, while the remaining 40% (considered “unknown samples”) were used for validation and prediction. Figure S2 (Supplementary Materials) depicts the obtained validation results. Classification rates were excellent, with 100% of samples correctly assigned for both calibration and prediction. A multiclass model was also considered to assign the set of samples under study simultaneously. Table 1 provides the sensitivity, specificity, and overall class prediction errors for the calibration and the cross-validation (CV). Overall, the obtained results were highly satisfactory, with sensitivity values for calibration and cross-validation higher than 90% and 85% (except for black tea which was 70%), respectively, and specificity values higher than 91% (for calibration) and 90% (for cross-validation). In addition, classification errors below 8% (for calibration) and 10.5% (for cross-validation) were, in general, obtained, with the only exception of black tea which showed a cross-validation classification error of 20%. The outstanding results (0% prediction error) obtained for the chicory group should be highlighted as chicory is the tea adulterant under evaluation.

The loading plot (Figure 3b) shows the polyphenolic variables responsible for the sample distribution and allows the identification of markers which are more discriminant of a specific sample group. It should be pointed out that for the four pairs of compounds coeluting, the peak area measured includes the contribution of both overlapping compounds, thus both of them may play a role in the sample discrimination. For instance, gallic acid and (+)-catechin + 4-hydroxybenzoic acid (at 280 nm) may be the more discriminant polyphenolic compounds in the white tea variety, while all the variables located at more negative LV1, i.e., rutin, *p*-coumaric acid, and apigenin, among others, are discriminant of green tea. In the case of chicory samples, caffeic acid could be proposed as a discriminant polyphenolic.

The good results obtained in the classificatory study demonstrate the good performance of the polyphenolic profiles (by monitoring only 17 flavonoids and phenolic acids at three acquisition wavelengths) for the classification of tea and chicory samples.

### 3.4. Adulteration Studies by PLS Regression

**Figure 4:**
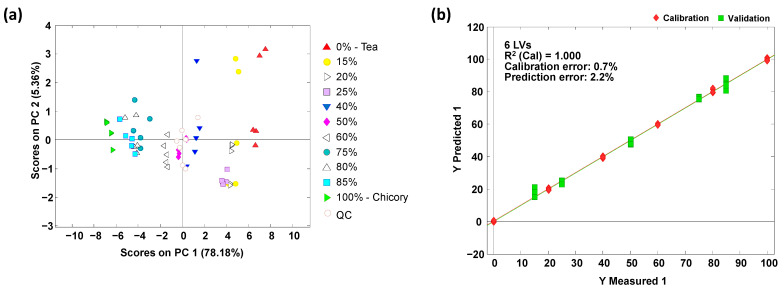
Green tea adulterated with chicory case. (**a**) PCA (PC1 vs. PC2) results showing the distribution of both calibration and prediction samples according to the chicory adulterant level. (**b**) PLS results showing the scatter plot of measured vs. predicted percentages of chicory adulterant.

Sample chemical descriptors based on the obtained polyphenolic profiles were also used for the detection of tea frauds and the quantitation of tea adulteration levels with chicory by PLS. For that purpose, the five possible adulteration cases, i.e., each tea variety adulterated with chicory, were addressed. Adulteration levels for both calibration and validation in PLS are described in Section 2.4. First, for each adulteration case under study, the polyphenolic profiles were subjected to PCA to see the distribution of the adulterations evaluated in the PC1 versus PC2 plot. As an example, the green tea adulterated with chicory case results are depicted in Figure 4a. Analogous results for the other four tea adulteration cases are provided in Figure S3 (Supplementary Materials). As shown in the PCA plots, pure samples (tea and chicory) are located at the extremes of the plot, while the different adulteration samples are distributed through the plot according to the adulterant percentages, from left to right across PC1. Then, PLS calibration models were built using the calibration set, and further predictions were made on the validation set. As can be seen in Figure 4b and Figure S3, the performance of the PLS calibration models was very satisfactory (results are summarized in Table 2). Good linearities (R^2^ values higher than 0.996) were obtained, with calibration, cross-validation and prediction errors below 2.0%, 4.2% and 3.9%, respectively. This is better than those previously reported by using non-targeted HPLC-UV or HPLC-FLD fingerprints [22], and even non-targeted FIA-MS fingerprints [23], demonstrating the feasibility of the targeted HPLC-UV polyphenolic profiles to assess tea authentication issues when adulterated with chicory.

As a proof of concept, the authentication of tea samples blended with teas of other varieties was also evaluated. For that purpose, the adulteration case based on a red tea extract blended with an oolong tea extract (considered as the adulterant), was also studied, and the obtained PCA and PLS data is provided in Figure S4 (Supplementary Materials). The obtained results were also very satisfactory, with good linearity (R^2^ 0.995), and calibration, cross-validation and prediction errors of 2.3%, 3.9% and 5.9%, respectively. These results confirm that the evaluated targeted HPLC-UV polyphenolic profiles also work as good chemical descriptors to authenticate teas when different tea varieties are blended.

In summary, the obtained results in this study confirm that HPLC-UV polyphenolic profiles are feasible and good chemical descriptors to characterize, classify and authenticate tea samples. In addition, data is useful to detect and quantify the levels of chicory in tea adulteration issues and to assess the authentication of teas when different varieties are blended.

## Figures and Tables

**Table 1:** Calibration and cross-validation multiclass predictions for the set of analyzed sample groups by PLS-DA.

Sample Class	Sensitivity (%)		Specificity (%)		Classification Error (%)	
	Calibration	Cross-Validation	Calibration	Cross-Validation	Calibration	Cross-Validation
Black tea	90	70	96	90	7	20
Green tea	95	90	97	94	4	8
Oolong tea	100	100	91	90	4.5	5
Red tea	95	90	98	99	3.8	5.5
White tea	90	85	94	94	8	10.5
Chicory	100	100	100	100	0	0

Sensitivity (%) = TP/(TP + FN) × 100, specificity (%) = TN/(TN + FP) × 100, accuracy (%) = (TP + TN)/TS × 100. TP, number of positive samples; TN, number of negative samples; FN, number of false negatives; FP, number of false positives; TS, total number of samples.

**Table 2:** Evaluation of the adulteration of tea with chicory by PLS using targeted HPLC-UV polyphenolic profiles as chemical descriptors.

Adulteration Case	LVs	Linearity (R^2^)	Calibration Error (%)	Cross-Validation Error (%)	Prediction Error (%)
Green tea	6	1.000	0.7	1.8	2.2
Black tea	4	0.998	1.5	2.9	2.7
White tea	6	0.999	1.1	2.2	3.6
Red tea	5	0.996	2.0	4.2	3.9
Oolong tea	5	0.999	0.9	1.7	1.7

## Data Availability

Data are available upon request to the authors.

## Supplementary Materials

The following supporting information can be downloaded at: https://www.mdpi.com/article/10.3390/foods12071501/s1, Figure S1: HPLC-UV (at 280 nm) separation of a polyphenolic and phenolic acid mixture at 20 mg L-1. Peak identification: 1, gallic acid; 2, protocatechuic acid; 3, quercetin; 4, (−)-epigallocatechin; 5, (+)-catechin; 6, 4-hydroxybenzoic acid; 7, vanillic acid; 8, caffeic acid; 9, syringic acid; 10, (-)-epicatechin; 11, *p*-coumaric acid; 12, ferulic acid; 13, sinapic acid; 14, rutin; 15, myrcetin; 16, apigenin; 17, kaempferol; Figure S2: Validation of the paired PLS-DA model of all tea varieties versus chicory when using targeted HPLC-UV polyphenolic profiles as the sample chemical descriptors. Red line means the separation threshold between classes. Filled and empty symbols correspond to the calibration and validation sets, respectively; Figure S3: PCA (PC1 vs. PC2) and PLS results of (a) black tea, (b) white tea, (c) red tea, and (d) oolong tea adulterated with chicory. Left plots: PCA scatter plots showing the distribution of both calibration and prediction samples according to the adulteration level. Right plots: scatter plots of measured vs. predicted percentages of chicory adulterant; Figure S4: (a) PCA (PC1 vs. PC2) and (b) PLS results of red tea extracts blended with oolong tea extracts (considered as the adulterant); Table S1: Detailed information about the analyzed tea and chicory samples; Table S2: Blends of each tea variety adulterated with chicory according to the percentage of chicory employed. X is a pooled tea variety sample (from 10 different tea samples). C1 to C3 represent the three different chicory samples (not pooled) employed as adulterants.
