# Supplementary material for: Targeted HPLC-UV Polyphenolic Profiling to Detect and Quantify Adulterated Tea Samples by Chemometrics
Source: Foods. 2023 Apr 3;12(7):1501. doi: 10.3390/foods12071501 (PMC10094304; doi:10.3390/foods12071501)
Supplement: Supplementary file 1 [file foods-12-01501-s001.zip › foods-2241852-supplementary.pdf]

SUPPLEMENTARY MATERIAL

Targeted HPLC-UV Polyphenolic profiling to detect and quantify adulterated tea samples by chemometrics

Thom Romers [1], Javier Saurina [1,2], Sònia Sentellas [1,2,3] and Oscar Núñez [1,2,3,*]

[1] Department of Chemical Engineering and Analytical Chemistry, Universitat de Barcelona, Martí i Franquès 1-11, E08028 Barcelona, Spain.
[2] Research Institute in Food Nutrition and Food Safety, Universitat de Barcelona, Av. Prat de la Riba 171, Edifici Recerca (Gaudí), E08921 Santa Coloma de Gramenet, Spain.
[3] Serra Húnter Fellow, Departament de Recerca i Universitats, Generalitat de Catalunya, Via Laietana 2, E08003 Barcelona, Spain.
* Correspondence: oscar.nunez@ub.edu

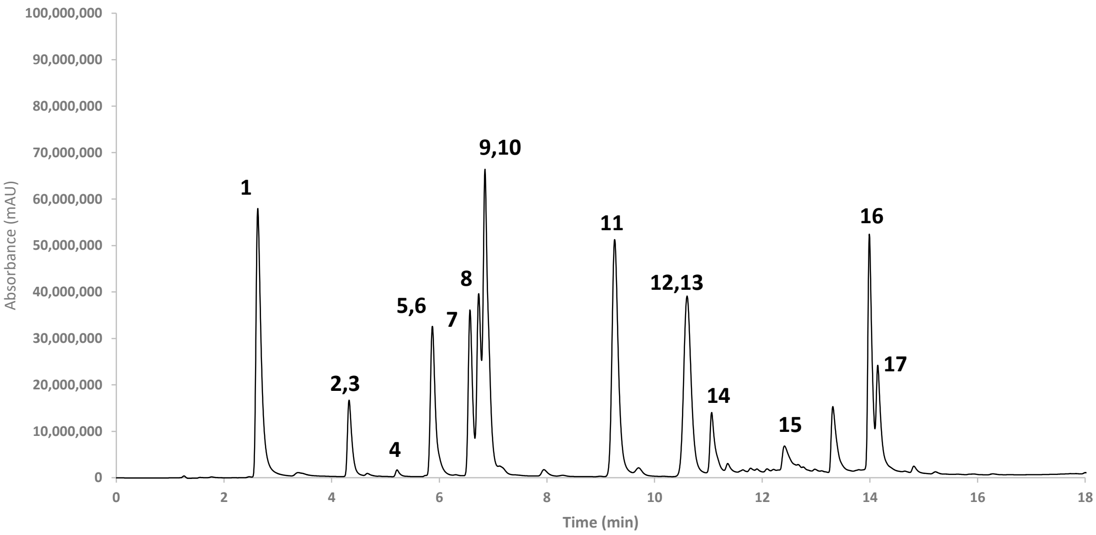

Figure S1. HPLC-UV (at 280 nm) separation of a polyphenolic and phenolic acid mixture at 20 mg L^{-1}. Peak identification: 1, gallic acid; 2, protocatechuic acid; 3, quercetin; 4, (-)-epigallocatechin; 5, (+)-catechin; 6, 4-hydroxybenzoic acid; 7, vanillic acid; 8, caffeic acid; 9, syringic acid; 10, (-)-epicatechin; 11, *p*-coumaric acid; 12, ferulic acid; 13, sinapic acid; 14, rutin; 15, myrcetin; 16, apigenin; 17, kaempferol.

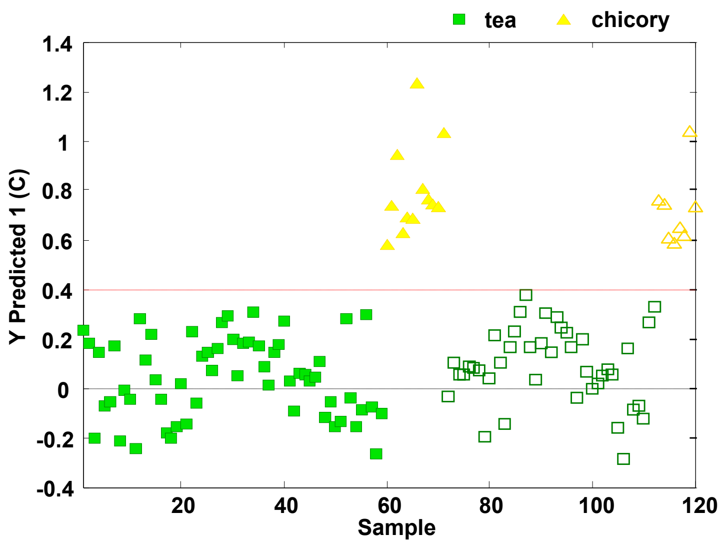

2 LVs
Classification rate (model): 100%
Classification rate (prediction): 100%

Figure S2. Validation of the paired PLS-DA model of all tea varieties versus chicory when using targeted HPLC-UV polyphenolic profiles as the sample chemical descriptors. Red line means the separation threshold between classes. Filled and empty symbols correspond to the calibration and validation sets, respectively.

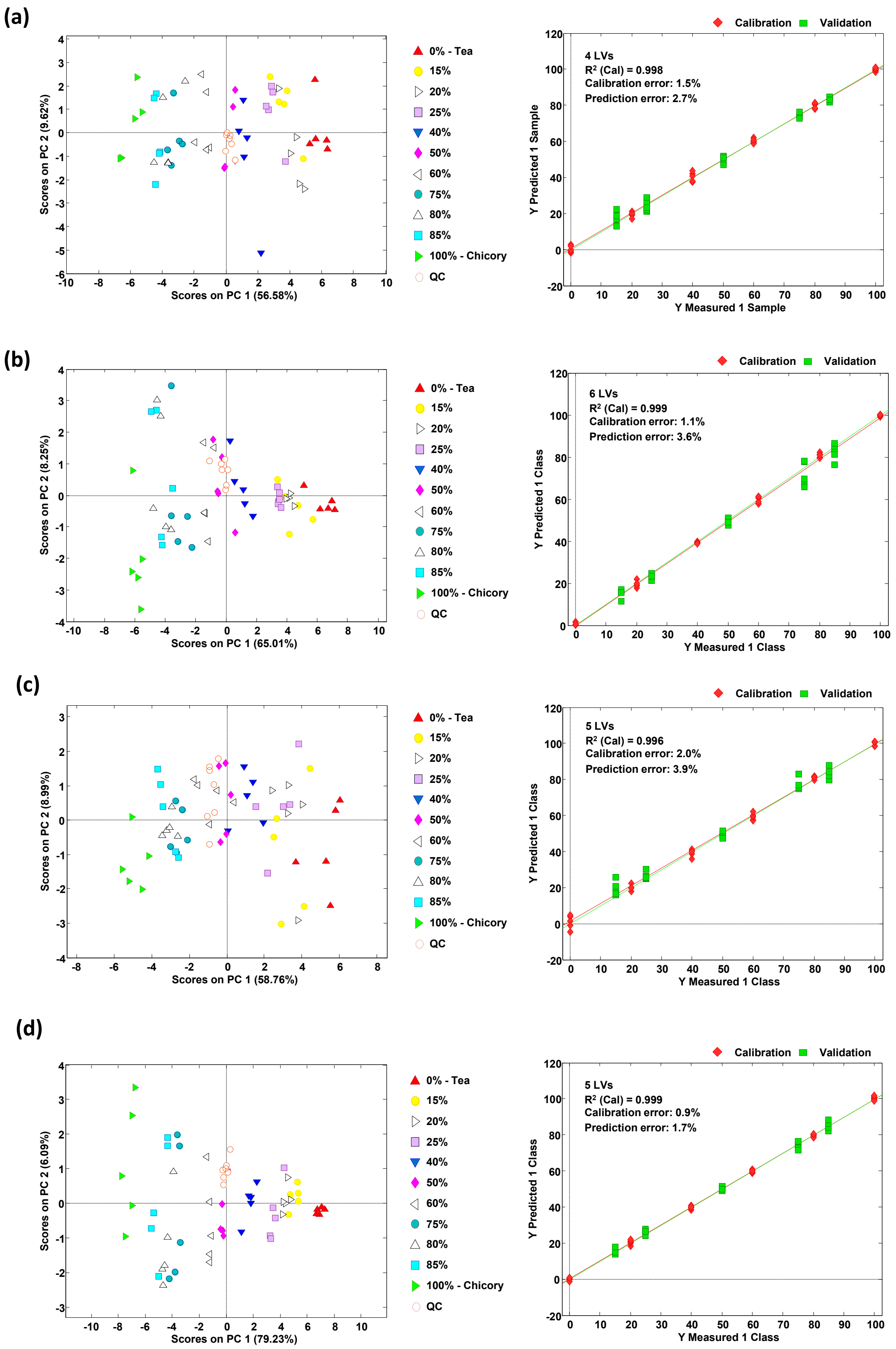

Figure S3. PCA (PC1 vs. PC2) and PLS results of (a) black tea, (b) white tea, (c) red tea, and (d) oolong tea adulterated with chicory. Left plots: PCA scatter plots showing the distribution of both calibration and prediction samples according to the adulteration level. Right plots: scatter plots of measured vs. predicted percentages of chicory adulterant.

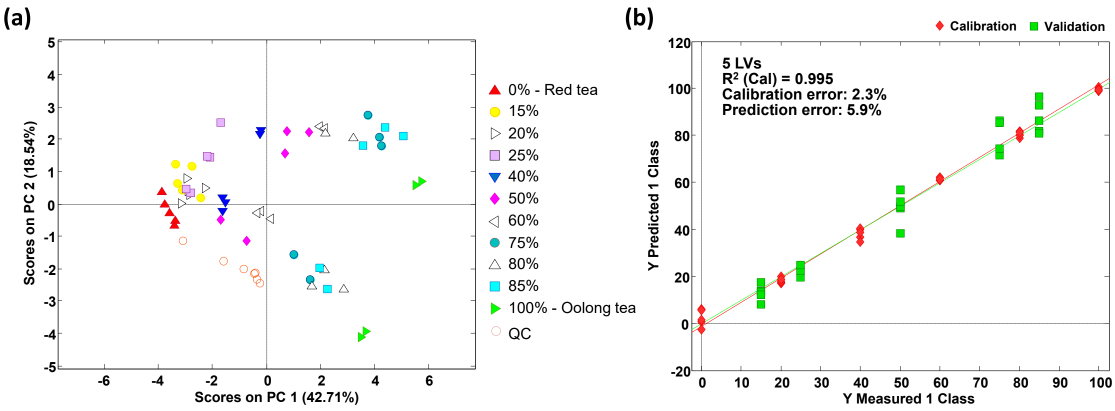

Figure S4: (a) PCA (PC1 vs. PC2) and (b) PLS results of red tea extracts blended with oolong tea extracts (considered as the adulterant).

Table S1. Detailed information about the analyzed tea and chicory samples.

Sample type	Commercial name	Production country	Number of samples (different lots)
Black tea	Ceylon black tea	Sri Lanka	2
	Irish blend	India	2
	Strong English breakfast	Sri Lanka	2
	Yunnan finest tippy, premium	China	2
	India Assam maud	India	1
	Kenya Marnyin	Kenya	1
	Darjeeling ringtong (second harvest)	India	1
	Darjeeling first flush (first harvest)	India	2
	Formosa tarry lapsang souchong	Taiwan	2
	Ceylon quality blend	Sri Lanka	1
	Ceylon Nuwara eliya	Sri Lanka	1
	Korakundah mountain tea	India	2
	Darjelling margaret's hope (first harvest, premium)	India	1
Green tea	Organic gunpowder	China	2
	Pi lo chun (premium)	Taiwan	2
	Sencha (Zhejiang)	China	2
	Lung ching	China	2
	Sencha (premium)	China	2
	Japan Bancha premium	Japan	2
	Japan gyokuro organic	Japan	2
	Mao Feng Jiangsu	China	2
	Assam Jamguri green	India	2
	Japan Kukicha organic	Japan	2
Oolong tea	Dong ding oolong	Taiwan	10
	Tie kuan yin	China	10
Red tea	Pu erh Royal (special fermentation)	China	5
	Pu erh Original	China	5
	Pu erh Imperial (manual harvesting)	China	5
	Pu erh Royal Palace	China	5
White tea	Pai Mu tan	China	5
	Silver needles (premium, artisanal production)	China	5
	Imperial Himalayan White Tea	India	5
	Vietnam Mao Feng Organic	Vietnam	5
Chicory	Chicory roots, Valley of Tea	Belgium	7
	Chicory roots, Health Embassy	England	7
	Chicory roots, Especias Pedroza	Spain	6

Table S2. Blends of each tea variety adulterated with chicory according to the percentage of chicory employed. X is a pooled tea variety sample (from 10 different tea samples). C1 to C3 represent the three different chicory samples (not pooled) employed as adulterants.

Adulteration level [%]	Tea	Chicory	Adulteration level [%]	Tea	Chicory
	X1			X1	C2
	X2			X2	C3
0	X3		60	X3	C1
	X4			X4	C2
	X5			X5	C3
	X1	C1		X1	C1
	X2	C2		X2	C2
15	X3	C3	75	X3	C3
	X4	C1		X4	C1
	X5	C2		X5	C2
	X1	C3		X1	C3
	X2	C1		X2	C1
20	X3	C2	80	X3	C2
	X4	C3		X4	C3
	X5	C1		X5	C1
	X1	C2		X1	C2
	X2	C3		X2	C3
25	X3	C1	85	X3	C1
	X4	C2		X4	C2
	X5	C3		X5	C3
	X1	C1			C1
	X2	C2			C2
40	X3	C3	100		C3
	X4	C1			C1
	X5	C2			C2
	X1	C3			
	X2	C1			
50	X3	C2			
	X4	C3			
	X5	C1			
